# Ecological genetics of range size variation in *Boechera* spp. (Brassicaceae)

**DOI:** 10.1002/ece3.1746

**Published:** 2015-10-15

**Authors:** John T. Lovell, John K. McKay

**Affiliations:** ^1^ Graduate Degree Program in EcologyC129 Plant SciencesColorado State UniversityFort CollinsCO80523‐1177USA; ^2^ Department of Integrative BiologyUniversity of Texas1 University Station, C0930AustinTX78712USA

**Keywords:** Diversity, evolutionary potential, growth rate, heterozygosity, phenotypic plasticity

## Abstract

Many taxonomic groups contain both rare and widespread species, which indicates that range size can evolve quickly. Many studies have compared molecular genetic diversity, plasticity, or phenotypic traits between rare and widespread species; however, a suite of genetic attributes that unites rare species remains elusive. Here, using two rare and two widespread *Boechera* (Brassicaceae) species, we conduct a simultaneous comparison of quantitative trait diversity, genetic diversity, and population structure among species with highly divergent range sizes. Consistent with previous studies, we do not find strong associations between range size and within‐population genetic diversity. In contrast, we find that both the degree of phenotypic plasticity and quantitative trait structure (*Q*_ST_) were positively correlated with range size. We also found higher *F*_ST*:*_ *Q*_ST_ ratios in rare species, indicative of either a greater response to stabilizing selection or a lack of additive genetic variation. While widespread species occupy more ecological and climactic space and have diverged at both traits and markers, rare species display constrained levels of population differentiation and phenotypic plasticity. Combined, our results provide evidence for a specialization–generalization trade‐off across three orders of magnitude of range size variation in the ecological model genus, *Boechera*.

## Introduction

The geographic range is one of the most fundamental ecological characteristics of a species (Darwin 1872; MacArthur 1972; Brown et al. 1996; Sexton et al. 2009), defining its conservation status, evolutionary dynamics, and interactions with the physical and biological environment (Gaston 1996; Geber 2011). Underlying the geographic range is a set of environmental conditions in which a species can maintain demographic stability (Hutchinson 1957; Brown 1984; Warren et al. 2008; but see Pulliam 1996). Under equilibrium conditions, the range is a spatial manifestation of the ecological niche (Hutchinson 1957; Pulliam 2000; Holt 2003). Therefore, range size can often be indicative of the diversity of environmental conditions that can be tolerated by a species (Brown 1984; Futuyma and Moreno 1988). Within most taxonomic groups, there exists a continuum between rare species (defined here as narrowly endemic species) with narrow ranges and those that are widespread across a large range of ecological conditions (Darwin 1872; Brown et al. 1996; Gaston 1996). As rare species are prone to the effects of environmental stochasticity and face the potential of extinction (Lande 1993; Payne and Finnegan 2007), understanding the processes underlying range size evolution is of great interest to conservation and evolutionary biologists (Kruckeberg and Rabinowitz 1985).

Rare species’ small geographic ranges may be a result of processes that are known to constrain evolution at the range margin, namely decreased evolutionary potential (Etterson and Shaw 2001; Etterson 2004; Pujol and Pannell 2008), low genetic diversity, or maladaptive gene flow (Angert and Schemske 2005; Blows and Hoffmann 2005; Angert et al. 2008; Gaston 2009; Eckhart et al. 2011). Range size may alternatively be a consequence of species’ physiological tolerances to environmental heterogeneity and stress. Finally, range margins may be stochastic, due to metapopulation dynamics, gene flow, or dispersal limitation, and independent of adaptive potential or physiological tolerances.

Many studies have shown that niche breadth is directly related to the putative diversity of phenotypes within a species (Brown 1984; Gaston and Spicer 2001; Slatyer et al. 2013; Sheth and Angert 2014), and the range of ecological conditions that permit a positive, or stable population growth rate (λ) across the landscape (Hutchinson 1957; Levins 1968; Spicer and Gaston 2009). Phenotypic diversity can be partitioned into the relative degree of heritable genetic population (or individual) differentiation and the extent of phenotypic plasticity. For example, widespread species with broad ecological niches may be characterized by highly differentiated populations (Bolnick et al. 2007; Nakazato et al. 2008; Ågren et al. 2013). Conversely, species with large ranges may represent a generalist strategy (Baker and Stebbins 1965). In this case, range size should be positively correlated with the capability of individuals to respond to diverse ecological conditions (plasticity) (Whitlock 1996; Pohlman et al. 2005; Kellermann et al. 2009). Indeed, Sheth and Angert (2014) recently demonstrated that widespread *Mimulus* species display a greater range of thermal tolerances than rare congeners. Finally, a trade‐off between plasticity and local‐scale specialization (jack of all trades, master of none) may drive patterns of niche breadth and the extent of the geographic range (Futuyma and Moreno 1988; Whitlock 1996; Caley and Munday 2003).

Closely related taxa that vary in range size provide an opportunity to investigate the evolutionary and ecological factors associated with rarity. For example, in an analysis of 34 species pairs, Gitzendanner and Soltis (2000) found weakly elevated genetic diversity in widespread species; however, 26% of the rare species were more diverse than their widespread congeners. These data, and other reviews (Karron 1987; Cole 2003), support the conclusion that elevated genetic diversity is sometimes, but not always, associated with increased range size. Alternatively, restricted range size may be due to shared physiological characteristics among rare species. Tests of this hypothesis in plants reveal that phenotypic divergence among taxa is not associated with range size, but is instead taxon and context dependent (Baskauf and Eickmeier 1994; Baskauf 2001; Richards et al. 2003; Lavergne et al. 2004; Pohlman et al. 2005). While these physiological and population genetic studies strive to document patterns associated with rarity, they lack information on quantitative genetic variation. Quantitative genetic variation and, in particular, the ratio of quantitative to neutral molecular genetic structure (e.g., *Q*_ST_‐*F*_ST_ comparisons) can shed light on patterns of historical adaptation (Whitlock 2008; Lamy et al. 2012). In the absence of dispersal limitation, the partitioning and extent of heritable genetic variation, plasticity, and molecular genetic structure (including gene flow) are thought to be the primary drivers of range limits (Angert and Schemske 2005; Pujol and Pannell 2008; Sexton et al. 2009; Slatyer et al. 2013); however, to date, there is little information on the underlying mechanisms that contribute to the evolution of range size.

Here, we conducted a combined quantitative and molecular genetic comparison across four species that span the variation in range size found within the genus *Boechera* (Brassicaceae). Among our study species were the two Colorado state‐listed (S2), imperiled members of the genus, *B. crandallii* and *B. vivariensis* (formerly *B. fernaldiana* ssp*. vivariensis*). This study marked the first genetic analysis and *ex situ* conservation efforts for these two species. We also included a widespread relative, *B. spatifolia,* and the well‐studied cosmopolitan species, *B. stricta*. Using these species, we directly assessed three predictions about the ecological and evolutionary causes and consequences of rarity. First, we predicted that individuals from widespread species should display elevated phenotypic plasticity relative to rare species. Second, we expected a greater level of population structure and total diversity in widespread species. Third, we expected rare species to be constrained to narrow distributions because of weak responses to selection and thus have lower levels of genetically based trait variation (heritability). Combined, these factors may limit the range of environments that permit demographic stability of rare species and may impede future adaptation to novel environmental conditions.

## Materials and Methods

### Study species

The genus *Boechera* is ideal for between‐species comparisons due to recent common ancestry, strong ecological divergence between species (Beck et al. 2012; Alexander et al. 2013), and the availability of diverse molecular and ecological tools (Lovell 2011; Rushworth et al. 2011). We quantified the geographic range of four *Boechera* species by summing the number of 0.1°latitude x 0.1°longitude (123 km^2^) grid cells that contain at least a single georeferenced herbarium specimen (e.g., Lloyd et al. (2002)) in DIVA‐GIS (www.diva-gis.org). Here, we classify *B. vivariensis* and *B. crandallii* as rare due to their restricted range sizes and listing in the Colorado rare plant index (http://www.cnhp.colostate.edu). The cosmopolitan *B. stricta* and moderately widespread *B. spatifolia* serve as the widespread species for all analyses. It is important to note that phylogenetic relationships among *Boechera* species are not clear, indicating a history of reticulate evolution and weak molecular divergence (Alexander et al. 2013), despite strong ecological and morphological divergence. As such, we have not attempted to construct phylogenetically independent contrasts.

**Figure 1:**
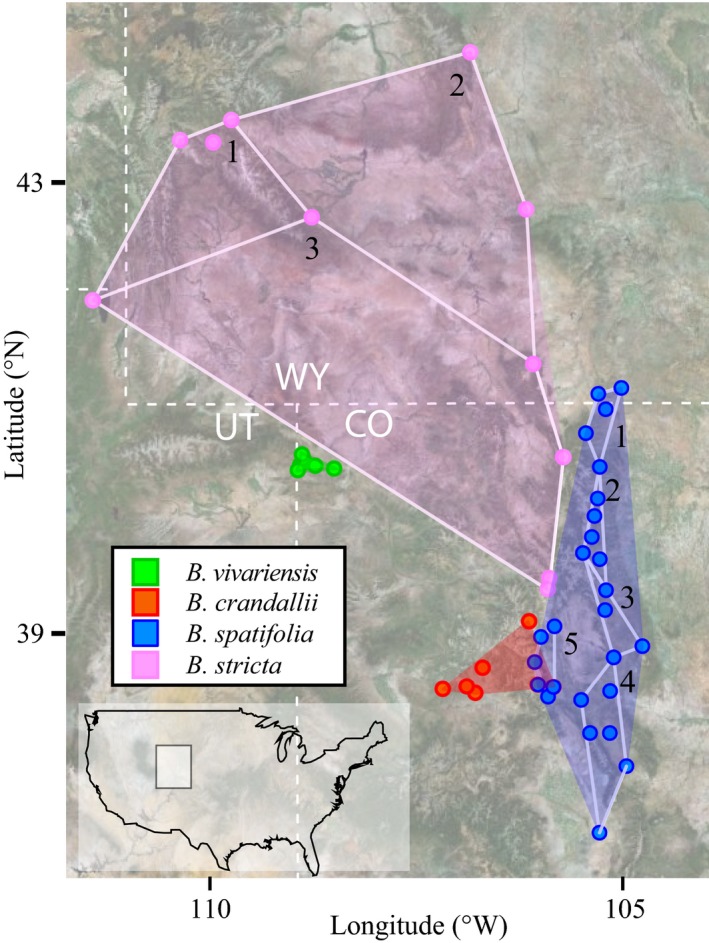
The sampled populations and genetic connectivity of the four species in Utah, Wyoming and Colorado, USA. The full distribution of all four species and the extent of the eight spatial subsampled populations groups (white polygons) are plotted.

The four study species are primarily sexual diploids (although apomictic and triploid individuals are known to exist at a low rate) that inhabit montane and semi‐arid environments of North America and exhibit winter annual or short‐lived perennial life histories (Roy 1995; Al‐Shehbaz 2003; Windham and Al‐Shehbaz 2006; Alexander et al. 2013). Molecular data from *B. crandallii* (Roy 1995), *B. stricta* (Song et al. 2006), and *B. spatifolia* (Lovell et al. 2014) indicate that self‐pollination is the dominant reproductive mode for each species. While such data are not available for *B. vivariensis*, similar floral morphology among these species suggests a comparable, low rate of outcrossing. Populations of the widespread species, *B. spatifolia* (*n*_pop_ = 26) (Lovell et al. 2014), and the rare species, *B. crandallii* (*n*_pop_ = 7) and *B. vivariensis* (*n*_pop_ = 6), were sampled across their entire geographic range. For the widespread species, *B. stricta* (*n*_pop_ = 17), sampling was conducted less densely, across a portion of the geographic range (Fig. 1). Seed collections were conducted across transects spanning the geographic extent of each population. To confirm that all sampled individuals were sexual diploids, we screened all seed families using the Flow Cytometric Seed Screen (FCSS: Matzk et al. (2000)) on a Partec PAII flow cytometer (Partec GmbH, Münster, Germany) following methods of Lovell et al. (2013a). FCSS data for *B. spatifolia* has been previously published (Lovell et al. 2014). All maternal families that produced a signature of apomixis or triploidy were excluded from this experiment.

### Phenotypic analysis

We planted four replicates (sibs) of each of 391 maternal families across the four species. The germination rate was 90%, resulting in 1401 plants: 186 *B. crandallii* (52 families from seven populations), 103 *B. vivariensis* (35 families from six populations), 717 *B. spatifolia* (193 families from 26 populations), and 394 *B. stricta* (111 families from 13 populations).

Plants were grown in 1” diameter RLC‐4 conetainers (Steuwe and Sons, Tangent, OR, USA) filled with Fafard 4P soil mix. Three seeds were placed directly on the soil and germinated following 14 days of cold stratification and thinned to one plant/conetainer. Growth conditions were designed to mimic those experienced by winter annual *Boechera* species: germination in early fall (days 1–14; 23/18°C, 12/12‐h day/night), growth during the fall (days 15–21; 18/8°C, 12/12‐h day/night), vernalization over the winter (days 22–54; 8/4°C, 8/16‐h day/night), and then growth in the spring (55–90; 23/18°C, 12/12‐h day/night). All plants were grown in a single Conviron ATC60 growth chamber at Colorado State University, Ft. Collins, CO, USA, and placed in racks of 200 plants. Rack and position within rack were completely randomized across the entire experiment. Each week, the location and orientation of each rack were randomly reassigned to reduce the effect of spatial variation in the chamber.

We measured eight traits: height, leaf area, and leaf number at 21 days postgermination (prevernalization); height, leaf area, and leaf number at 55 days postgermination (postvernalization); and internode distance (leaf number/height at 55 days) and leaf size (leaf area/leaf number at 55 days). Height and leaf number were measured directly, while leaf area was calculated by extracting the canopy area from photographs taken directly above the plant. Measures of height and leaf area were also taken at two additional time points, 28 and 63 days postgermination, in order to measure growth rate in winter and spring conditions, respectively. Image processing was completed in Photoshop CS5.1 (Adobe Corporation, San Jose, CA, USA), and analysis of leaf area was conducted in ImageJ (http://rsbweb.nih.gov/ij/). We chose these traits because leaf morphology and vegetative rosette architecture vary considerably across differently adapted populations in other herbaceous plant species (McKay et al. 2001; Leinonen et al. 2009).

As many of the phenotypic traits were correlated, we calculated quantitative genetic statistics from principal component scores. Each individual was assigned the mean of the first three principal component scores, weighted by the %variance explained by each axis (PC1 = 56.9%, PC2 = 21.0%, PC3 = 12.0%). This weighted mean PCA score is the phenotypic value used for all quantitative genetic analyses. To calculate variance components, we fit a random effects linear model using the *lmer* function in the R package “lme4” (Bates et al. 2014). The model contained two factors: population of origin and seed family nested within population. From this model, we extracted the among‐population (*V*_POP_), among‐family (*V*_FAM_), and residual (*V*_ERR_) variance components and calculated total variance (*V*_TOT_) as the sum of these three components. We then calculated the genetic variance partitioned among populations (*Q*_ST_ = *V*_POP_/(*V*_FAM_ + *V*_POP_) and estimated broad‐sense heritability as the proportion of total variance partitioned among families, within population (*H* ^2^ = *V*_FAM_/*V*_TOT_). *Boechera* exhibits a primarily self‐pollinating mating system; however, outcrossing is known to occur. Therefore, our families are primarily inbred selfed progeny, with a small proportion of half sibs. As such, we followed Lee and Mitchell‐Olds (2013) and present our estimates of *H* ^2^ and *Q*_ST_ as the uncorrected (for family structure) ratio of among‐population nonresidual variance and among‐family variance to total variance, respectively. We recognize that these data might diverge from heritability estimates via controlled pedigrees.

We measured plasticity as the change in sequentially measured traits across environmental conditions. This measure of plasticity, also referred to as acclimatization, or flexibility in animals (Bradley 1978), provides a direct estimate of the magnitude of response by genotypes to changing environmental conditions (Pelletier et al. 2007). Individuals were grown in fall and then in winter temperature and photoperiod conditions. In each condition, we calculated growth rate of leaf area (*GR*_LA_) (Lovell et al. 2013b) and stem elongation (*GR*_SE_ = ln(height_*t*2_ *) −* ln(height_*t*2_)) / (days_*t*2_‐days_*t*1_). For each growth rate trait, we calculated breeding values of plasticity as the family‐level mean of the absolute value of the difference between environments (Falconer and Mackay 1996; Van de Pol and Wright 2009). Plasticity was compared among species in a mixed effects linear model where species was the only fixed effect and population was the random effect.

### Genetic analysis

DNA was extracted from lyophilized leaf tissue using the ChargeSwitch gDNA plant kit (Invitrogen Corp. Carlsbad, CA, USA). We followed PCR and genotyping protocols optimized by Beck et al. (2012) for 15 SSR markers known to amplify well across most *Boechera* species. Three‐primer set multiplexed PCR and genotyping were conducted on all samples. Primers were constructed with “FAM” and “HEX” labeled dyes and genotyped on an ABI 3130xL Genetic Analyzer at the Colorado State University proteomics and metabolomics facility. Alleles were called using the GeneMapper v4.0 (Applied Biosystems). PCR conditions and primer information can be found in Table S1. Three loci gave null alleles in *B. crandallii*. One of these was also null in *B. vivariensis*. These alleles were coded as null and incorporated into the analysis. Individuals with missing data for >4 SSRs or signatures of duplication were excluded. Following inclusion of the null alleles and exclusion of individuals with poor amplification, our genotyping success was >98%.

We calculated six summary statistics using the *basic.stats* function in the R package “hierfstat” (Goudet 2005). These include *H*_*O*_ (observed heterozygosity), *H*_*S*_ (average within‐population gene diversity, a.k.a. expected heterozygosity), *H*_*T*_ (total gene diversity), *D*_ST_ (*H*_*T*_ − *H*_*S*_, average absolute magnitude of differentiation among populations), *F*_ST_ (*D*_ST_/*H*_*T*_, proportion of total variation partitioned among populations), and *F*_IS_ (1− *H*_*O*_/*H*_*S*_, inbreeding coefficient). Finally, we implemented an analysis of connectivity among populations through spatial population genetic graphs in the R package “popgraph” (Dyer and Nason 2004) using an edge retention α = 0.05. Using the function *edge.betweenness.community* in the R package “igraph” (Csardi and Nepusz 2006), we calculated modularity, or the maximum modularity score of all community structures, which defines the degree to which the species‐level network can be broken into distinct groups.

### Statistical comparisons among species

All statistical comparisons were conducted in the R Environment for Statistical Computing 3.0.2 (R Core Team 2013). To infer the statistical significance of observed differences between rare and widespread species, we calculated bootstrapped confidence intervals for all relevant statistics.

This was accomplished for quantitative genetic diversity estimates by extracting variance components from the random effects linear model fit by the *lmer* function in the “lme4” R package (Bates et al. 2014). The linear random effects model was bootstrapped 1000 times using the *bootMer* function that accompanies *lmer*. Phenotypic plasticity bootstrapping was accomplished in an identical manner to the quantitative genetic statistics, except that the fixed effect estimates of species were extracted from the linear mixed effect model. Bootstrapped estimates (1000×) of molecular genetic indices were calculated using a custom R function (*boot.basicstats*, https://github.com/jtlovell/rarity.analyses) that rarefies then bootstraps the genetic data. For each statistic, we compared bootstrapped estimates of each species. Significant differences were defined as any case where the 95% confidence interval of the bootstrapped distributions of one species did not overlap with that of another species.

To remove any confounding role that spatial distribution or sample size may introduce into our analyses, we conducted a spatially explicit population subsampling approach by extracting sets of populations from the widespread species that mimicked the distribution of the rare species. We then recalculated the plasticity, molecular, and quantitative genetic statistics for the subsampled populations. To compare these subsamples to the rare species values, random single subsampled population groups were drawn (20×) from each of the widespread species and compared to the rare species using an identical linear model described above. The median *P* and *r* ^2^ values were reported from these distributions.

Finally, we conducted linear regressions of the mean bootstrapped value for each species and the log_10_ range size (km^2^). This was accomplished with the *lm* function in R.

## Results

The four species exhibit the extremes of geographic range variation in *Boechera*. Area occupied by each species in our study region (Fig. 1) was 1113 km^2^ (*B. vivariensis*;* n*_pop_ = 5, *n*_family_ = 48), 3339 km^2^ (*B. crandallii*;* n*_pop_ = 6, *n*_family_ = 46), 27080 km^2^ (*B. spatifolia*;* n*_pop_ = 21, *n*_family_ = 151), and 98924 km^2^ (*B. stricta*;* n*_pop_ = 11, *n*_family_ = 95). Additionally, geographic range size was highly correlated with the range of environmental variation underlying the geographic distribution. For example, the range of growing season precipitation (*BIO18*) was on average 4× greater across the widespread species’ than the rare species’ distributions (Table S2).

Microsatellite‐derived genetic distances were relatively equivalent among species, without evidence of phylogenetic species pairs (Fig. S1). This is consistent with a recently published phylogenetic analysis of *Boechera* (Alexander et al. 2013). Summary data from the 15 SSR loci can be found in Table 1.

**Table 1:** Summary of population genetic diversity: For each species and locus, we calculated the total number of alleles (*A*), % of individuals with missing data (%NA), and observed heterozygosity (*H*_*o*_)

Locus	*B. vivariensis*			*B. crandallii*			*B. spatifolia*			*B. stricta*		
	%NA	*A*	*H*_*O*_	%NA	*A*	*H*_*O*_	%NA	*A*	*H*_*O*_	%NA	*A*	*H*_*O*_
a1	0.00	2	0.11	0.00	2	0.00	5.70	2	0.02	0.79	2	0.00
bf11	0.00	1	0.00	0.00	1	0.00	3.63	3	0.15	0.00	4	0.00
bf18	0.00	1	0.00	0.00	1	0.00	1.04	2	0.00	3.15	4	0.00
b6	0.00	2	0.03	0.00	1	0.00	0.52	11	0.09	3.94	1	0.00
a3	0.00	3	0.14	0.00	1	0.00	0.00	1	0.00	9.45	3	0.20
bf20	0.00	6	0.49	1.96	4	0.13	5.18	8	0.18	0.79	5	0.02
c8	6.38	5	0.08	0.00	2	0.00	6.74	3	0.18	14.96	6	0.01
bf9	17.02	4	0.21	0.00	3	0.12	1.04	6	0.13	3.15	12	0.02
bf19	6.38	17	0.77	0.00	1	0.00	12.44	5	0.14	3.94	10	0.06
bdru266	61.70	10	0.33	0.00	1	0.00	0.52	17	0.18	2.36	12	0.04
ice3	0.00	12	0.50	1.96	6	0.10	6.22	7	0.18	0.79	7	0.01
ice14	2.13	3	0.05	0.00	1	0.00	3.63	2	0.00	0.00	3	0.00
e9	0.00	5	0.42	0.00	3	0.06	1.04	5	0.13	3.15	12	0.03
bf3	4.26	20	0.77	1.96	1	0.00	0.00	8	0.13	3.94	11	0.00
bf15	8.51	3	0.35	3.92	5	0.23	0.00	5	0.17	2.36	4	0.01

### Rarity was not correlated with neutral genetic diversity

**Figure 2:**
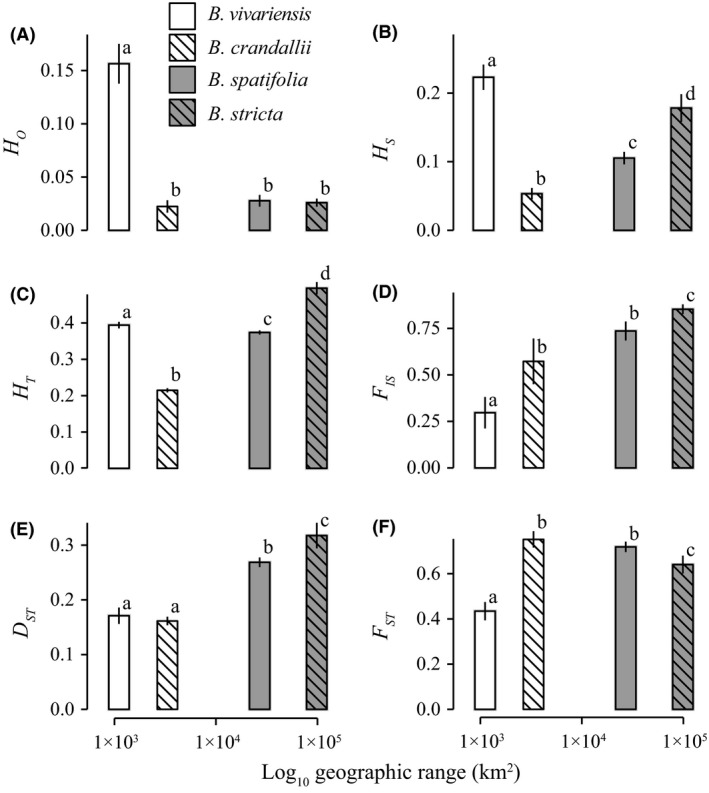
Molecular genetic diversity and structure of rare and widespread *Boechera* species. The mean statistical value (+/‐ the standard deviation of 1000 bootstraps) is reported. Grey bars indicate widespread species, while white bars indicate rare species. Species are indicated by either empty (*B. vivariensis*,* B. spatifolia*), or cross‐hatched fill (*B. crandallii*,* B. stricta*). Other figures follow this labeling pattern.

The most narrowly distributed species, *B. vivariensis*, displayed the highest (*P *<* *0.05) levels of within‐population genetic diversity (*H*_*O*_, *H*_*S*_, Fig. 2A,B; *A*_*e*_, Fig. S2a). The other rare species, *B. crandallii*, had significantly lower average within‐population gene diversity (*H*_*S*_) and effective number of alleles (*A*_*e*_, Fig. S2b) than any other species and the lowest observed heterozygosity (*H*_*O*_) albeit not significantly lower than the widespread species. Interestingly, the two rare species *B. vivariensis* and *B. crandallii* exhibited the highest and lowest levels of within‐population genetic diversity, respectively. As such, range size was not significantly correlated with these measures of genetic variation.

We also measured total gene diversity (*H*_*T*_) and the proportion of variance within populations found within individuals (*F*_*IS*_). Consistent with our within‐population diversity estimates, the rare species *B. vivariensis* and *B. crandallii* displayed elevated and the lowest *H*_*T*_ values, respectively (Fig. 2C). Across the four species, *F*_IS_ showed a positive correlation (albeit not significant) with geographic range size, where the highest and lowest *F*_IS_ values were found in the most widespread and rarest species, respectively (*n *=* *4, *r* ^2^ = 0.63, *P *>* *0.1; Fig. 2D).

### Quantitative and molecular structure co‐varies with range size

Measures of molecular genetic connectivity among populations varied considerably among species. While all populations of *B. vivariensis* were highly interconnected (modularity = 0.024), populations of *B. crandallii* (0.26), *B. spatifolia* (0.72), and *B. stricta* (0.31) displayed at least 10× greater modularity (Fig. S2a‐d). As such, the degree of network connectivity was greater in rare than in widespread species. Elevated genetic structure among samples was also manifest in widespread species by significantly greater *D*_ST_ (among‐population differentiation; Nei 1973; Fig. 2E), but not *F*_ST_ (proportion of among‐population gene diversity) levels (Fig. 2F).

**Figure 3:**
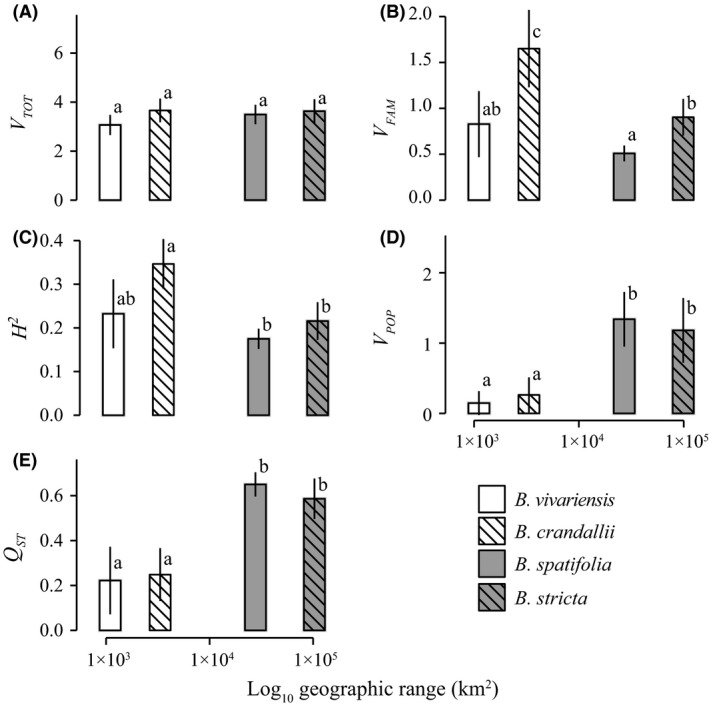
Quantitative genetic diversity and structure of rare and widespread *Boechera* species. The mean statistical value (+/‐ the standard deviation of 1000 bootstraps) is presented.

Total phenotypic variance (*V*_TOT_) was similar across all four species (Fig. 3A); however, variance among families, within populations (*V*_FAM_; Fig. 3B) and the proportion of total within‐population variance partitioned among families (*H* ^2^; Fig. 3C) were lowest in the widespread species, *B. spatifolia,* and highest in the rare species, *B. crandallii*. Both widespread species displayed significantly lower *V*_FAM_ and *H* ^2^ than *B. crandallii*. In general, widespread species had lower values of both among‐family statistics than rare species. We observed the opposite pattern for among‐population variance (*V*_POP_) and the proportion of nonresidual variance partitioned among populations (*Q*_ST_). The widespread species displayed significantly elevated values for both statistics (Fig. 3D,E).

**Figure 4:**
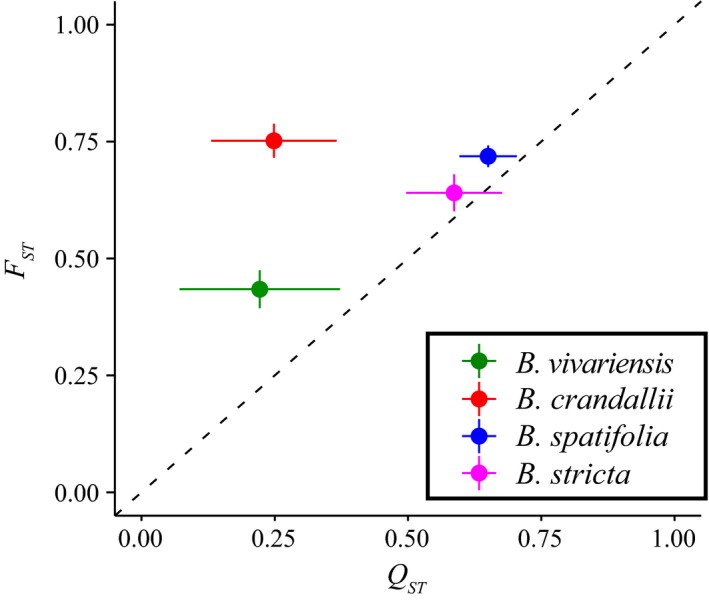
Correlations between molecular and quantitative genetic partitioning among the four species. Mean *Q*_ST_ and *F*_ST_ (+/‐ standard deviation of 1000 bootstrap resamples) are plotted, color‐coded by species. Species’ ratios are significantly different from 1:1 when the standard deviation does not overlaps the dashed line.

Finally, we examined the correlation between the partitioning of neutral molecular and quantitative genetic variation (Fig. 4). Such *F*_ST_/*Q*_ST_ ratios can shed light on the relative effect of drift and selection among populations of a species. While a *F*_ST_/*Q*_ST_ ratio of 1 is expected if populations are evolving by drift, strong responses to stabilizing selection and low additive genetic variance or directional selection could shift this ratio up or down, respectively. Both widespread species had *F*_ST_/*Q*_ST_ ratios that were not significantly different from one. However, the two rare species, *B. vivariensis* and *B. crandallii*, had significantly greater ratios than expected by drift (1.96, 3.02, respectively; Fig. 4).

### The effects of spatial subsampling on estimation of genetic parameters

The observed differences among species with different geographic ranges could either be 1) due to intrinsic attributes of the species – these traits would not change when widespread species were subsampled, or 2) affected by the geographic range of sampling and not a species‐specific attribute – these traits would remain correlated with range size within species across subsampled population groups. To test between these hypotheses, we spatially subsampled the widespread species (*B. spatifolia* and *B. stricta*) into five and three population groups, respectively, that covered geographic areas ranging from 236 to 35941 km^2^ (see the white polygons in Fig. 1) containing 5 *≤* *n ≤ *7 populations. These distributions reflected those of the rare species.

**Figure 5:**
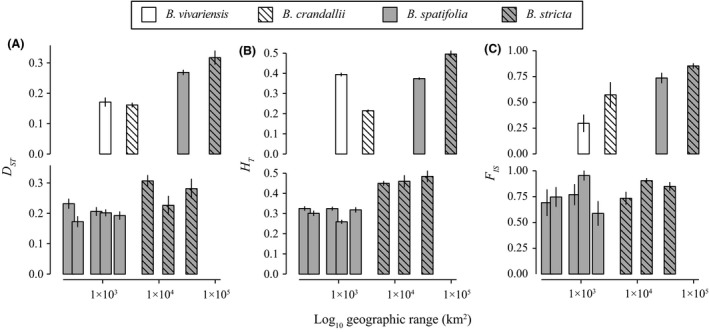
Comparison of statistics derived from full‐species samples (top row) and spatially subsampled distributions of the widespread species (bottom row). Due to denser population sampling, we were able to define five subsampled groups for *B. spatifolia*, but only three for *B. stricta*. Three statistics are presented for each species and subsample: *D*_ST_ (panel A), *H*_*T*_ (panel B), *F*_IS_ (panel C).

Both *D*_ST_ and *F*_IS_ remained marginally greater in both widespread species. This consistent effect across all subsamples and main samples was evidenced by as strong a positive correlation with range size (*D*_ST_: *n *=* *4, median *r* ^2^ = 0.82, median *P *=* *0.11; *F*_IS_: *n *=* *4, median *r* ^2^ = 0.88, median *P *=* *0.06; Figs 5 and S3) as among the full sampled species (Fig. 2E). As such, these traits, and not the other population genetic statistics (Fig. S3), were intrinsic attributes of each species. For the quantitative genetic statistics, subsampling generally reduced among‐population statistical estimates; however, within‐population estimates were robust to subsampling (Fig. S3).

### Elevated phenotypic plasticity is associated with widespread species

**Figure 6:**
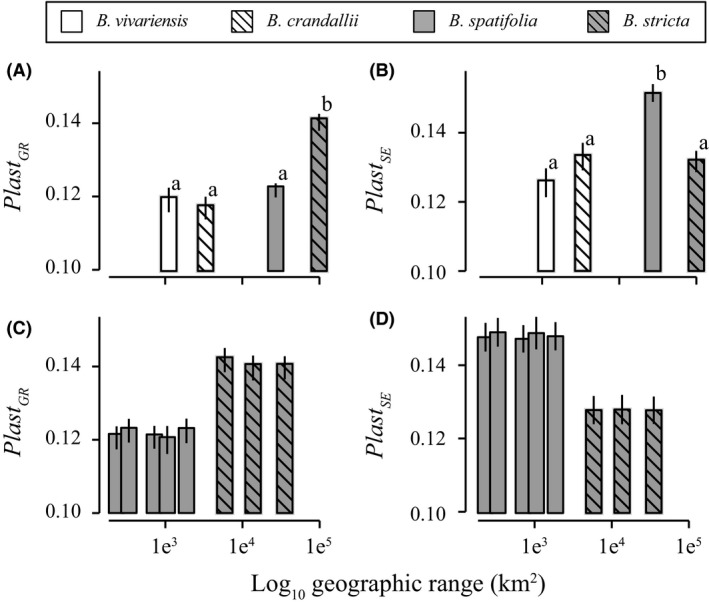
Phenotypic plasticity of the full samples (A, B) and spatial population subsampling of the widespread species (C, D). Means (+/‐ the standard deviation of 1000 bootstraps) are presented.

The widespread species *B. spatifolia* and *B. stricta* displayed the highest values of the two measures of growth rate plasticity, leaf area (*Plast*_LA_), and stem elongation (*Plast*_SE_), respectively (Fig. 6A,B). However, for each plasticity measure, one of the widespread species was not statistically different from either of the rare species. When an average plasticity measure is taken, there was a strong correlation between plasticity and range size across the full sampled distributions (*n *=* *4, *r* ^2^ = 0.92, *P *=* *0.04), where species with broader geographic distributions had greater plasticity of growth rate. However, this correlation does not remain significant for each trait independently. Interestingly, the *F*_ST_/*Q*_ST_ ratios were negatively (albeit not significantly) correlated with mean plasticity estimates (*n* = 4, *r *=* *−0.81, *P *>* *0.1).

Finally, we tested whether the strong effect of among‐species range size on plasticity persisted within species across subsampled populations. The full‐species level of plasticity was maintained in all subsampled distributions regardless of the range of subsampled populations (*n *=* *4, median *r* ^2^ = 0.01, median *P *>* *0.1; Fig. 6C,D). As such, there is no evidence that our estimates of plasticity were affected by our sampling scheme.

## Discussion

Our results were consistent with the hypothesis that widespread species have broader ecological niches (Slatyer et al. 2013) and should therefore display elevated phenotypic variation and plasticity (Sheth and Angert 2014) relative to rare species. The increased phenotypic variation found in widespread species was due to both strong spatial structure (>3× increase in among‐population variance relative to rare species) and increased phenotypic plasticity. However, our neutral molecular analysis ran contrary to the hypothesis that rare species should have decreased genetic diversity. Molecular genetic diversity was not significantly different between rare and widespread species across all species and diversity indices. While total diversity was similar among species types, there was generally less structuring between populations of rare species than between populations of common species.

From an evolutionary perspective, rare species may have narrower ranges because of an inability to adapt to novel conditions at the range margin (Sexton et al. 2009). Therefore, we hypothesized that rare species should have limited within‐population heritable genetic variation of potentially adaptive traits. Our data did not support this hypothesis. Rare species generally had as much or greater within‐population quantitative genetic trait variation and heritability than widespread species, factors that may lead to increased responses to selection.

### Molecular diversity within and across populations

Rarity holds many definitions, including extreme endemism and less geographically restricted but sparsely inhabited ranges. In her seminal review of rarity, Rabinowitz (1981) argued that rare species could not be classified by a single demographic pattern. Distinguished by opposing values of population connectivity, census size, and spatial extent, *B. crandallii* and *B. vivariensis* represent opposite ends of the spectrum of narrowly endemic rare species. Despite having the narrowest range, *B. vivariensis* maintained the largest and densest populations of all study species (J.T. Lovell personal observation). Also, its highly connected habitat and small spatial distribution may contribute to increased genetic exchange and decreased modularity among populations. Due to drift, measures of molecular diversity, such as *F*_ST_, *H*_*e,*_ and *H*_*O*_, may be correlated with effective population size (*N*_*e*_); therefore, it is possible that the demography of these rare species drove the observed within‐population diversity patterns. The presence of broadly different molecular genetic patterns of variation in the two rare species demonstrates that rare species can exhibit widely varying population genetic attributes.

Across all molecular genetic diversity indices, there were no consistent associations between genetic diversity and range size (Figs 2 and S3). While total genetic diversity was higher, but not significantly so, in widespread species, we observed the opposite pattern for within‐population diversity. As expected, within‐population measures of genetic diversity were robust to the geographic extent of population sampling (Fig. S3); however, there was a significant effect of subsampling on the species‐level diversity index, *H*_*T*_, where subsampled distributions displayed decreased diversity relative to the full population sample. It is important to note that *B. spatifolia* and *B. stricta* exhibited little isolation by distance (IBD, data not shown). In highly structured species with high IBD, a larger reduction in diversity could be expected following subsampling. Many comparisons of molecular diversity have utilized a wider distribution and higher sample size for widespread species relative to the rare congener (e.g., Song and Mitchell‐Olds (2007); Takahashi et al. (2011)). In these cases, it is possible that species‐level diversity may be confounded by sampling scheme.

### Genetic structure, plasticity, and correlations with range size

The widespread species studied here exhibited geographic ranges 1–3 orders of magnitude larger and occupied considerably more heterogeneous ecological and climactic habitats than the rare species (Table S2). We hypothesized that these widespread species would exhibit elevated total phenotypic diversity compared with their more rare congeners. Generally, our results do not support this hypothesis (Figs 3 and 6). The total amount of variation was nearly identical among species. Instead, the partitioning of variance and the extent of plasticity distinguished rare from widespread species. A much greater proportion of variance was found among populations (*Q*_ST_) in widespread species than of rare congeners. This might be caused by lower historical responses to selection, decreased intensity of diversifying selection and/or increased gene flow.

Plasticity can have opposing effects on adaptation (Whitlock 1996; Price et al. 2003; Ghalambor et al. 2007) and population persistence at the range margin. A high degree of plasticity will expand the amount of suitable habitat (Baker and Stebbins 1965; Pohlman et al. 2005; Sheth and Angert 2014), and studies have shown that increased plasticity can be beneficial when invading new sites (Loomis and Fishman 2009), surviving stress (Heschel et al. 2004), or persisting through changing environmental conditions (Chevin and Lande 2011). However, plasticity may be nonadaptive (Relyea 2002), reducing the local fitness. By calculating differences in growth rate across environmental conditions, we found that, in general, genotypes of widespread species had significantly greater plasticity of either leaf area growth rate or stem elongation rate than those of rare species (Fig. 6). It is important to mention that plasticity was not significantly elevated in widespread species across plasticity measures, but only for one trait in each widespread species. It is possible that the faster and more extreme phenotypic adjustments of widespread species would permit homeostasis of fitness across more diverse environments, leading to increased niche breadth. However, future studies that measure fitness and physiology across diverse conditions are needed to directly address this hypothesis.

### Trade‐offs between specialization and generalization and implications for range size evolution

Understanding the relative contribution of local adaptation and plasticity to niche breadth variation is a foundational issue in the study of range size evolution (Whitlock 1996; Kirkpatrick and Barton 1997). If low levels of plasticity and poor adaptation to local conditions characterize rare species, then extinction should soon follow (Lande 1993; Chevin et al. 2010). However, several studies have demonstrated significant local adaptation in rare species (McKay et al. 2001; Song and Mitchell‐Olds 2007). Here, we assessed two contrasting hypotheses about the quantitative genetic structure of widespread species relative to rare species: (1) Widespread species have a highly plastic physiology that is amendable to many environments, and (2) widespread species demonstrate high levels of local adaptation, leading to higher quantitative genetic trait structure.

We observed a positive correlation between population structure and plasticity. Widespread species had both greater plasticity and greater quantitative genetic structure than rare species. Additionally, widespread species showed decreased among‐family trait variance. This lack of heritable genetic variance is an indicator of decreased potential to adapt to future conditions. However, it is possible that standing genetic variation in widespread species has been disproportionally exposed to, and purged by, historical directional selection.

Rare species had significantly greater *F*_ST_/*Q*_ST_ ratios. Elevated *F*_ST_/*Q*_ST_ indicates either poor responses to selection or strong responses to stabilizing selection (Whitlock 2008; Lamy et al. 2012). Interestingly, both rare species are locally abundant within their preferred habitat. However, the widespread species exhibit patchy or dispersed population distributions. As such, the widespread species seem to be relatively more poorly adapted to extremely local conditions than rare species but more successful across the landscape. It is possible that a combination of high levels of structure, potentially caused by historical responses to selection, and elevated phenotypic plasticity have permitted widespread species to colonize a range of habitats, at the cost of excelling within a single environment. Alternatively, rare species may have responded to local stabilizing selection pressures, which improved local persistence and fitness at the expense of broader ecological tolerances – one of the costs of phenotypic plasticity.

### Additional considerations for the use of molecular and quantitative genetic tools in the study of rarity

Summary statistics describing molecular polymorphism within populations and species are routinely used to make decisions about conservation of rare species. However, the majority of studies comparing rare and widespread species do not find strong associations between genetic diversity and range size. Our analyses also find genetic diversity to be a poor correlate of rarity. While well suited for characterization of population structure and relatively inexpensive to obtain, neutral genetic diversity estimates may not provide an adequate way to test the hypothesis that rare species lack the potential to adapt to novel environmental conditions (Frankham et al. 1999; Vitt and Havens 2004), nor do they provide direct evidence of local adaptation or the potential for future adaptive evolution (McKay et al. 2001). Population genetic analysis can provide valuable information about the demographic history of a species, while measures of quantitative genetic variation directly address adaptive potential and may provide insight into the evolutionary causes and consequences of rarity.

As rare species were characterized by decreased population structure and phenotypic plasticity, they may be more exposed to the short‐term effects of climate change and anthropogenic landscape modification. However, elevated within‐population heritable variation may improve their response to selection and permit an evolutionary response to future environmental change.

## Conflict of Interest

The authors declare no conflict of interests.

## Data Accessibility

All genotype data will be electronically archived in Dryad (http://datadryad.org/). Additional data and statistics can be found in the accompanying supplemental material.

## Supporting information


**Figure S1**. Principal component analysis derived from 15 SSR loci demonstrated the amount of divergence between the species.
**Figure S2.** Individually scaled close‐ups of the four species’ sampling distributions are plotted in the context of a molecular genetic network derived from the 15 SSR loci.
**Figure S3**. Molecular and quantitative genetic diversity of rare and widespread *Boechera* species.
**Table S1.** List of primer names, labels and sequences used in the SSR analysis. PCR was conducted with 5‐PRIME HotStart Master Mix (Gaithersburg, MD, USA) in 12ml reactions using the following PCR conditions: initial denaturation (95C, 120sec), [denaturation (94C, 30sec) annealing (53C, 90sec), extension (65C, 60sec)], number of cycles (25), final extension (65C, 30mins).
**Table S2.** Environmental diversity underlying the geographic range of each species.
