# Supplementary material for: Ecological genetics of range size variation in Boechera spp. (Brassicaceae)
Source: Ecol Evol. 2015 Oct 15;5(21):4962–75. doi: 10.1002/ece3.1746 (PMC4662302; doi:10.1002/ece3.1746)
Supplement: Supplementary file 1 — Figure S1. Principal component analysis derived from 15 SSR loci demonstrated the amount of divergence between the species. Figure S2. Individually scaled close‐ups of the four species’ sampling distributions are plotted in the context of a molecular genetic network derived from the 15 SSR loci. Figure S3. Molecular and quantitative genetic diversity of rare and widespread Boechera species. Table S1. List of primer names, labels and sequences used in the SSR analysis. PCR was conducted with 5‐PRIME HotStart Master Mix (Gaithersburg, MD, USA) in 12ml reactions using the following PCR conditions: initial denaturation (95C, 120sec), [denaturation (94C, 30sec) annealing (53C, 90sec), extension (65C, 60sec)], number of cycles (25), final extension (65C, 30mins). Table S2. Environmental diversity underlying the geographic range of each species. [file ECE3-5-4962-s001.docx]

Supporting Information:


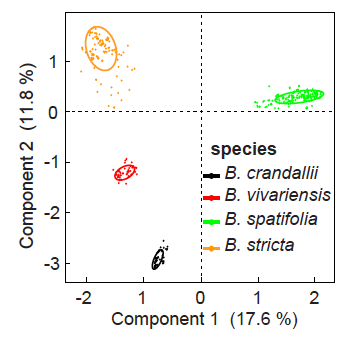


**Figure S1**. Principal component analysis derived from 15 SSR loci demonstrated the amount of divergence between the species. PCA axis 1 and 2 scores for each individual were plotted by species with the associated 95% confidence ellipse.


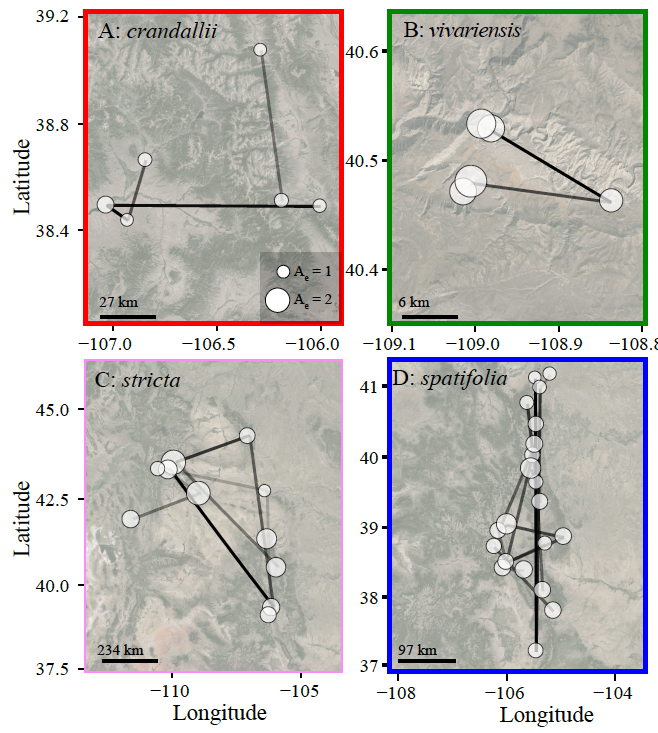


**Figure S2.** Individually scaled close-ups of the four species’ sampling distributions are plotted in the context of a molecular genetic network derived from the 15 SSR loci. Nodes in the network represented the population locations and were size-scaled by the effective number of alleles (*A_e_*). Edges connecting nodes that exceeded a threshold of α = 0.05 were plotted as black lines and weighted by connectivity (more opaque edges are stronger connections).


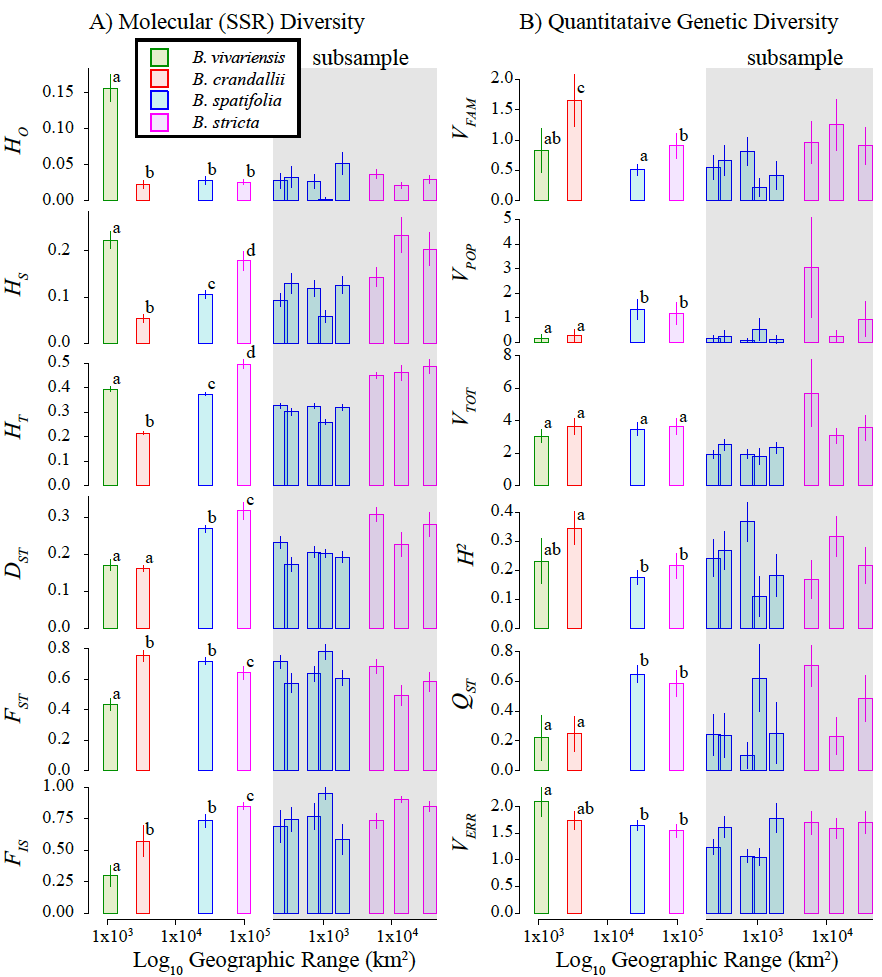


**Figure S3**. Molecular and quantitative genetic diversity of rare and widespread *Boechera* species. The mean statistical value (+/- the standard deviation of 1000 bootstraps) is reported. In each panel, the four full-species statistics were represented by the four color-coded bars on the left side. The eight colored bars overlaying the gray background, represented the five *B. spatifolia* (blue) and three *B. stricta* (pink) subsampled mean and bootstrapped standard deviations for each statistic. The statistics are grouped into molecular genetic diversity (panel A) and quantitative genetic diversity (panel B) .

Supplemental Figure Captions, Tables and Table Captions

| Multiplex Set | Primer Name | Label | Forward Primer | Reverse Primer |
| --- | --- | --- | --- | --- |
| 1 | I3 | 6-FAM | GACTAATCATCACCGACTCAGCCAC | ATTCTTCTTCACTTTTCTTGATCCCG |
|  | B20 | HEX | TTCTCGGGAAAGTAATGAGGAG | GCAAATCTGACCAATGCAAG |
|  | A1 | 6-FAM | GTCTATTCGAGGACGCC | AGGTTGGGTAGGTGAAG |
| 2 | B11 | 6-FAM | TCCTCCATTGTAGAGCAGAGC | CCATTGCTTAAACCCTAAACC |
|  | I14 | HEX | TCGAGGTGCTTTCTGAGGTT | TACCTCACCCTTTTGACCCA |
|  | C8 | 6-FAM | TTCCGGGTATCATTCCTAG | GTTGTAAGTTCTTTCTCAG |
| 3 | B9 | 6-FAM | AAACACATTCCCGTCAGCTC | TTGATTGAATCCTGCGTTTG |
|  | B18 | HEX | AACCTCCCAAGATTCGCTTC | TTCGCCATTGTTGTGATTTG |
|  | E9 | 6-FAM | AGGAAAGGACAAAAGACATG | GCTTCCATGGAAGGAGACCC |
| 4 | BF3 | 6-FAM | TTTTTAGACAGTAGTGGCTGTGAG | ACTTCGTTCCAGGCTCGTC |
|  | BF19 | HEX | ACCGCATTGGTGTTGTGTC | ATAACGGACGCGACCAAAG |
|  | B6 | 6-FAM | GCAAAAGATCTTCATGGGAC | TGCCATTTCTTTCCCTAGTG |
| 5 | BF15 | 6-FAM | CAGCATCTCCTTTGGGTTTG | ACTTGCTCCTTTGCATGACC |
|  | B266 | HEX | TTTAATTTGTGCGTTTGATCC | CAAAATCGCAGAATGAGAGG |
|  | A3 | 6-FAM | AGCTTTGTTTGCAATGGAG | GTGAGAATAATATTGACC |

**Table S1**: List of primer names, labels and sequences used in the SSR analysis. PCR was conducted with 5-PRIME HotStart Master Mix (Gaithersburg, MD, USA) in 12ml reactions using the following PCR conditions: initial denaturation (95C, 120sec), [denaturation (94C, 30sec) annealing (53C, 90sec), extension (65C, 60sec)], number of cycles (25), final extension (65C, 30mins).

| Species | *vivariensis* | *crandallii* | *spatifolia* | *stricta* |
| --- | --- | --- | --- | --- |
| Range (km2) | 1113 | 3339 | 27080 | 98924 |
| *bio1* | 21 | 90 | 116 | 147 |
| *bio2* | 22 | 47 | 63 | 82 |
| *bio3* | 2 | 3 | 14 | 13 |
| *bio4* | 753 | 1592 | 2509 | 2875 |
| *bio5* | 37 | 125 | 131 | 182 |
| *bio6* | 11 | 84 | 127 | 148 |
| *bio7* | 47 | 97 | 131 | 155 |
| *bio8* | 97 | 81 | 242 | 316 |
| *bio9* | 10 | 174 | 213 | 277 |
| *bio10* | 28 | 100 | 119 | 167 |
| *bio11* | 10 | 73 | 115 | 119 |
| *bio12* | 86 | 379 | 588 | 843 |
| *bio13* | 5 | 34 | 64 | 91 |
| *bio14* | 6 | 26 | 38 | 45 |
| *bio15* | 6 | 36 | 57 | 50 |
| *bio16* | 22 | 84 | 161 | 229 |
| *bio17* | 18 | 97 | 127 | 157 |
| *bio18* | 25 | 86 | 198 | 237 |
| *bio19* | 18 | 140 | 189 | 280 |
| *alt* | 471 | 1488 | 1911 | 2256 |

**Table S2**: Environmental diversity underlying the geographic range of each species. The range (maximum value – minimum value) of each of the 19 BIOCLIM variables (bio1-19) and elevation above sea level (alt) were presented for each species.
